# Editorial: Veterinary rhinology and emerging insights in olfaction

**DOI:** 10.3389/falgy.2025.1668340

**Published:** 2025-08-08

**Authors:** Melissa Singletary, Cynthia M. Otto

**Affiliations:** ^1^ Department of Anatomy, Physiology and Pharmacology, College of Veterinary Medicine, Auburn University, Auburn, AL, United States; ^2^ Canine Performance Sciences Program, College of Veterinary Medicine, Auburn University, Auburn, AL, United States; ^3^ Department of Clinical Sciences & Advanced Medicine, School of Veterinary Medicine, University of Pennsylvania, Philadelphia, PA, United States

**Keywords:** mammalian olfaction, detection canine, animal special senses, veterinary smell, odor detection

**Editorial on the Research Topic**
Veterinary rhinology and emerging insights in olfaction

## Introduction

Rhinology is the human specialty focused on the treatment of diseases of the nose and sinuses, including olfactory disorders. Veterinary rhinology, historically, has focused on diseases affecting the nasal and sinus anatomy and pathology affecting respiratory function, but has not had the tools to investigate conditions affecting the sense of smell or even normal olfaction. Increased significance is being placed on olfaction due to its relevance in biological function and its applications with a high societal impact placed on the canine species. Many studies in olfaction use rodent models, which can serve as a translational model for other species; though increasingly, applied studies are conducted within the species of interest. In this Research Topic, Veterinary rhinology and emerging insights in olfaction, six manuscripts address the function, evaluation, environmental influences, and applications in olfaction in animals with an emphasis on working animals.

### Function

The underlying physiological processes that result in odor perception and recognition are complex and not fully understood. Functional anatomical studies highlighting airflow dynamics associated with odorant delivery to the olfactory regions have provided insights into the mechanical transportation of odorants in multiple animal models (1, 2). The 1991 breakthrough discovery of the G-protein coupled receptor repertoire for the olfactory receptor genes led to a series of discoveries in the olfactory signal transduction cascade (3). Despite these groundbreaking discoveries and decades of study to follow, the ability to predict odor perception from physicochemical properties is still relatively elusive (4, 5). As the field aims to understand the molecular mechanisms involved in olfaction, new insights are shared which support the presence of an odor-evoked potassium current that mediates olfactory responses in a rodent model (Hagerty et al.).

### Evaluation

This special issue spans topics from basic neurophysiology to applied olfaction. Fundamentally, functional veterinary rhinology relies on techniques and methods that support olfactory system evaluation. The first requirement of a standardized evaluation is to understand the odor source, dynamics, and presentation. The second component is to provide a standardized assessment. Sloan et al. present detailed information on the principles that guide the chemical properties of odor and odor availability. Further providing context to strategies in odor manipulation for training versatility in detection canines with applicability to a variety of odor-detection-based platforms. Maughan et al. provide the potential use and application of a universal detector calibrant in the field of detection canines within development, training, and evaluation. The versatility and value of a standardized odor include application for odor detection thresholds (Aviles-Rosa et al.) and maintenance of search motivation (6–8). Further studies in working detection animals and untrained animals may provide clinical and operational evaluations for animal health and performance.

### Environmental influences

The function of the olfactory system is modulated by local microenvironmental influences and macroenvironmental factors. In working odor-detection animals, the performance outcome of identifying the source of the odor depends on (1) the presence or absence of odor, (2) the accessibility of the odor and (3) the animal's odor detection threshold. Reduced detection efficiency can result from limited odor accessibility inherent to the target or presentation of materials (e.g., low vapor pressure, low diffusivity, or low dispersal (9) or limited biosensor (animal) capability due to internal or external factors (e.g., threshold (10, 11), experience (12, 13), temperature (14–16). In a canine physical exertion model, the odor detection thresholds were impacted by the intensity and duration of the exercise. A decrement in performance was observed after 20 min of moderate intensity exercise. These findings provide proof-of-concept that there is an interaction between intensity and duration which impacts olfactory performance (Aviles-Rosa et al.).

### Application

Harnessing olfaction for applications that benefit society has been a practice for millennia as dogs hunted alongside humans using olfaction in prey localization, and carrier pigeons provided long-distance communication. Modern applications of animal olfaction have advantageously used a variety of species for rapid mobile detection capabilities in safety, security, conservation, public health, and an ever-expanding list of novel applications. The capability of an intelligent biosensor with an evolutionarily fine-tuned olfactory sensory system seems endless with the right selection and preparation. Novel proof-of-concept studies in biological detection fields highlight an area of this unique application of dogs in complex biological processes for the improvement of human and animal health. A study by Ramos et al. demonstrated a high level of sensitivity and specificity of a cohort of detection dogs to *Staphylococcus aureus* biofilms. The study utilized a bacterial culture-based training approach, which is accessible and subject independent and demonstrated successful generalization to lacrimal tear fluid samples collected from infected mammals, including humans and rabbits, suggesting an odor signature which may be detectable across sample types (Ramos et al.). Further research in this area may open avenues for the use of non-invasive odor-based diagnostics. Another study by Kiiroja et al. evaluated the sensitivity of dogs to detect changes in hormonal fluctuations associated with stress and post-traumatic stress disorder. The study conducted with two pilot dogs demonstrated support for the olfactory capacity to identify a stress-associated odor(s) in human breath samples. Generalization differences between the two dogs across stress types suggest differing sample constituents driving individual odor learning, which may align in a divergence to either the sympathetico-adreno-medullar*y* axis or the hypothalamic-pituitary-adrenal axis) (Kiiroja et al.). These studies provide novel applications in odor-based detection and highlight the complex and dynamic capabilities of the mammalian olfactory system.

### Overall summary

The field of olfactory neuroscience is concerned with one of the earliest evolved senses, the sense of smell. The interplay of external environmental factors, regional micro-environmental conditions, and the highly integrated neural processing of the olfactory pathway lends to its complexity and capability. This research topic incorporated the latest developments in veterinary rhinology from basic to applied research and captured emerging insights in olfaction. Working animals provide a unique window into understanding mammalian olfaction and its applications in the safety, health, and security of humans and animals. Further exploration is needed in the understanding and application of olfaction, as we've only scratched the surface.
